# Ensemble Machine Learning Model for Accurate Air Pollution Detection Using Commercial Gas Sensors

**DOI:** 10.3390/s22124393

**Published:** 2022-06-10

**Authors:** Wei-In Lai, Yung-Yu Chen, Jia-Hong Sun

**Affiliations:** 1 Institute of Applied Mechanics, National Taiwan University, Taipei 106, Taiwan; r08543039@ntu.edu.tw; 2 Department of Mechanical and Materials Engineering, Tatung University, Taipei 104, Taiwan; yychen@ttu.edu.tw; 3 Department of Mechanical Engineering, Chang Gung University, Taoyuan 333, Taiwan

**Keywords:** AI, RNN, IoT, ensemble model, model retraining, CO gas detecting, O_3_ gas detecting, NO_2_ gas detecting

## Abstract

This paper presents the results on developing an ensemble machine learning model to combine commercial gas sensors for accurate concentration detection. Commercial gas sensors have the low-cost advantage and become key components of IoT devices in atmospheric condition monitoring. However, their native coarse resolution and poor selectivity limit their performance. Thus, we adopted recurrent neural network (RNN) models to extract the time-series concentration data characteristics and improve the detection accuracy. Firstly, four types of RNN models, LSTM and GRU, Bi-LSTM, and Bi-GRU, were optimized to define the best-performance single weak models for CO, O_3_, and NO_2_ gases, respectively. Next, ensemble models which integrate multiple single weak models with a dynamic model were defined and trained. The testing results show that the ensemble models perform better than the single weak models. Further, a retraining procedure was proposed to make the ensemble model more flexible to adapt to environmental conditions. The significantly improved determination coefficients show that the retraining helps the ensemble models maintain long-term stable sensing performance in an atmospheric environment. The result can serve as an essential reference for the applications of IoT devices with commercial gas sensors in environment condition monitoring.

## 1. Introduction

Effectively monitoring and controlling air quality has become an important issue of concern to the public today. Air pollution significantly impacts human health, ranging from mild chronic respiratory symptoms to acute respiratory infections, exacerbating pre-existing heart and lung diseases [1,2]. Even if people only expose themself to mild air pollution, it will shorten their lives [3]. Thus, people living in urban areas or nearby industrial areas demand information on atmospheric air quality because they have a higher risk of exposure to elevated air pollution [4].

To acquire the air quality information, government and environmental protection agencies have started to set up fixed-site air quality monitoring stations in various regions. These monitoring stations have accurate instruments to regularly monitor air quality in the environment, analyze the concentration of pollutants, and provide information to the public for reference [5,6]. However, building more fixed-site air quality monitoring stations is difficult due to terrain limitations and the high cost of setting up and maintaining the stations. The monitoring data provided by the stations are relatively sparse and therefore cannot meet the increasing demand for air quality information. Therefore, various techniques have been proposed to improve the spatial density of air quality information, such as the detection of short-term air quality campaigns by the mobile laboratory [7], interpolation by mathematical models [8], and detection by various new low-cost sensors [9,10]. With more air quality information, more services, such as forecasts and real-time alarms, can be provided to the public. The low-cost sensors are popular with people today, because the components are available and affordable in the market. People who care about air pollution can obtain a direct onsite measurement themselves, and, furthermore, environmental protection agencies have also started to adopt low-cost sensors in areas without a monitoring station to obtain supplementary air quality information [11].

Although low-cost sensors are economical and easy to set up, some performances still stand to be improved, including their accuracy, cross-sensitivity, reproducibility, and reliability [12]. To enhance the detection of sensors, many researchers have focused on developing new sensing materials. Chemically modified SnO_2_ nanosurfaces using metals or other metal oxides showed highly selective sensing materials for CO, NH_3_, H_2_S, and NO_2_ gas molecules [13]. PbS quantum dots/TiO_2_ nanotube arrays possess a good response towards NH_3_ gas at room temperature [14]. Furthermore, gas sensor arrays based on MEMS gas sensor platforms, consisting of different nano-sized and metal oxide semiconductor (MOS) particles, were developed to detect CO, NOx, and NH_3_; their gas-sensing characteristics in the binary mixed-gas system were investigated [15]. The new sensing materials can make gas sensing more accurate, but these low-cost sensors have not fully met all the practical needs.

On the other hand, artificial intelligence (AI) technology has rapidly developed in recent years and has been successfully applied in many fields. Thus, especially in terms of machine learning, AI technology was also combined with gas sensors for more accurate detection. An artificial neural network (ANN) model was used with a sensing array that had four quartz crystal microbalance (QCM) devices to distinguish the type of organic vapors [16]. An array that contained six ZnO-based sensors was combined with an ANN model to recognize concentrations of H_2_, CH₄, and CO gases [17]. A surface acoustic wave (SAW) sensor coated with a functionalized polymer detected harmful vapors, and an ANN pattern-recognition model was implemented to recognize vapor types [18]. The research mentioned above show that combining a gas sensor array with an ANN model can identify mixed gases’ compositions and concentrations in a well-controlled laboratory. This result is consistent with the report that sensors’ performance was concluded to be satisfactory under a range of specific conditions [11]. ANN models were further applied with sensors in the field with consideration of temperature, humidity, wind speed, and pressure. Field calibration of low-cost commercial sensors in detecting NO, CO, and CO_2_ gases was studied. The result showed that ANN is the most effective method among linear regression, multiple linear regression, and ANN [19]. Another CO electrochemical sensor was also calibrated with an ANN model by considering temperature and humidity [20]. Thus ANN has good results in in-field gas classification and concentration identification.

Recurrent neural network (RNN) is another deep learning method often used to solve sequence problems due to its gated unit design [21,22]. RNN models contain different types of model, Long Short-Term Memory (LSTM) [23], Gated Recurrent Unit (GRU) [24], Bi-directional Long Short-Time Memory (Bi-LSTM) [25], and Bi-directional Gated Recurrent Unit (Bi-GRU). Since time characteristics accompany gas-sensing data, RNNs are expected to deal with time-dependent gas concentrations and other interfering factors. The LSTM model was used to predict air pollution concentrations with MOS gas sensors in Amravati and Bengaluru, India [26]. Different RNNs also estimated the gas concentrations considering temperature, flow rate, and negative pressure, and the LSTM model has higher prediction accuracy [27]. In another measurement conducted using MOS gas sensor arrays, considering temperature, relative humidity, and absolute humidity, the LSTM model had higher accuracy than the ANN model in the concentration recognition of a gas mixture [28]. The results show that the RNN model can effectively analyze the gas concentration data when it has interference factors; with a better model performance, improving the accuracy of low-cost sensors is feasible.

In fact, both the ANN and the RNN have made good progress in in-field gas sensing. ANN first made feasibility in gas classification and concentration detection; RNN further processed the sequence measured concentration data and interference factors to improve accuracy. Moreover, it is noticeable that sensors may have inconsistent performance in different measurement scenarios. For example, performance can vary significantly in different areas due to different environmental conditions [10,26,29]. In practical applications, the long-term correlation between low-cost gas sensors and reference instruments is not stable, mainly due to the change in field temperature and humidity [10]. Thus, the gas-sensing calibration technology still has the problem of generalizability.

The ensemble model is a recent solution to the bottleneck of deep learning, which improves the prediction performance of a single model by training multiple single models and combining their prediction results [30,31]. Ensemble machine learning has been used widely in various fields, such as face recognition, target tracking, and bioinformatics [32,33,34,35]. On the other hand, the current research on gas sensing with machine learning are all based on a single individual model, and thus an ensemble model has the potential to improve gas-sensing performance.

Therefore, this paper aims to study the development of ensemble models to monitor in-field gas concentrations with low-cost commercial gas sensors. The generalizability of the trained model to be used with sensors in different environmental conditions was also tested. First, we collected the concentrations of CO, O_3_, and NO_2_ gases and the atmospheric conditions with homemade IoT devices for the following model training. The preprocessing of data included outlier detection and normalization. Second, four types of RNN model, LSTM, GRU, Bi-LSTM, and Bi-GRU, were introduced; a loss function and an evaluation function were also defined for training and optimizing a single RNN model. In the third part, the four types of optimized RNN single model were presented. Then, an ensemble model containing static models, i.e., the optimized RNN single models and a dynamic model, was composed and trained. For better generalizability of the model, a retraining procedure for the ensemble model was processed to make the model more flexible to adapt to various environmental conditions and improve the long-term sensing performance. Finally, the discussion and conclusion were presented.

## 2. Data Preparation

### 2.1. IoT Device Designing and Data Collection

**Figure 1:**
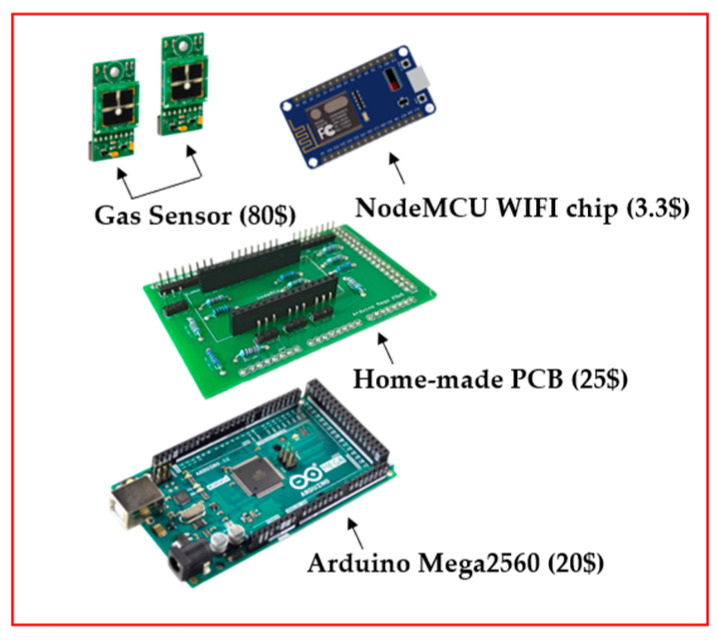
Component assembly diagram of IoT device.

To detect gaseous pollutants in the environment, the authors developed a low-cost wireless gas-sensing device in the study. Figure 1 shows the low-cost Internet of Things (IoT) [36] device used in the research. It consists of four components: a gas sensor, a NodeMCU WIFI chip, a Homemade PCB, and a Arduino Mega2560. The gas sensor is a commercial component sold on the market, which detects the concentration of target gases, including CO, O_3_, and NO_2_. The sensors also detect the temperature and humidity in the atmosphere simultaneously. The Arduino Mega2560 stores the data detected by the sensors, while the NodeMCU WIFI chips upload the data stored by the Arduino Mega2560 to the cloud via the WIFI devices. The homemade PCB, designed by the author, integrates the interface of various electronic components, which reduces the occupied space and increases the stability of the component. The typical total cost of this device is 200 USD.

The low-cost IoT devices were then set up at a monitoring station established by the Environmental Protection Administration (EPA), Taiwan. This monitoring station is located in Guting Elementary School, Taipei city. Air quality measurements were taken using the instruments in the station (HORIBA APMA360 for CO, ECOTECH ML9810 for O_3_, and ECOTECH ML9841 for NO_2_) and then published on the EPA website. Our IoT devices were placed in the instrument shelter of the Guting monitoring station to ensure that the device’s environment was not disturbed by illumination fromthe sun, rain, and ground radiation. Then, the IoT devices measured the ambient temperature, humidity, and gas concentrations in the atmosphere continuously for three months. The data collected from 5 January to 23 March 2021, were used to develop machine learning models, including training, validation, and first testing; the data recorded from 23 March to 14 April were used for more testing to examine the models’ long-term stability.

**Figure 2:**
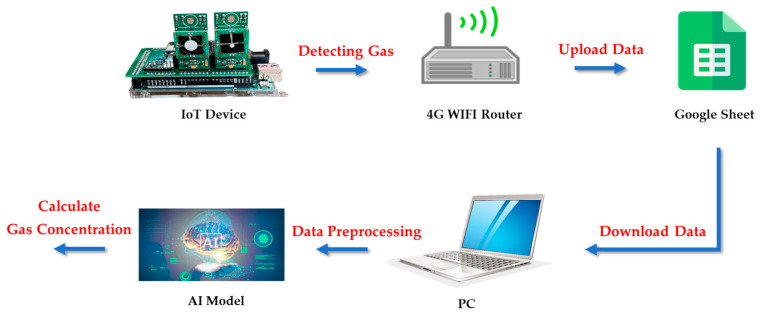
Schematic diagram of the architecture of the Internet of Things.

The IoT architecture divides into the perception, network, and application layers. As shown in Figure 2, the hardware of the perception layer is a low-cost IoT device responsible for detecting target gas concentrations and transmitting the detected concentration data to the network layer. The hardware of the network layer is the 4G WIFI router, which is responsible for receiving the information transmitted by the perception layer and uploading the received data to Google Cloud through the device. The hardware of the application layer is a personal computer. It obtains the information transmitted by the perception layer from the Google cloud and performs data preprocessing on the acquired data. The preprocessing process includes outlier cleaning, data normalization, and feature selection. Thus, the preprocessed atmosphere data transmits to the trained AI model using the IoT architecture for further gas concentration calculation.

The commercial gas sensors used in the mentioned IoT device have different performance characteristics. Table 1 shows their detecting ranges and resolutions, and the cross-sensitivity information of each gas sensor is listed in Table 2. The manufacturer of these gas sensors is SPEC [37]. This study refers to each air pollutant’s annual average concentration values in 2020, as shown in Table 3 [38]. The average annual concentration of CO gas in 2020 was 0.35 ppm, indicating that the average annual concentration of CO gas was greater than the resolution of the CO gas sensor (0.1 ppm in Table 1). Thus, the CO gas sensor can effectively respond to the target gas in the environment at this concentration level. The annual average concentration of O_3_ gas in 2020 was 30.9 ppb, indicating that the annual average concentration of O_3_ gas was slightly higher than the resolution of the O_3_ gas sensor (20 ppb). The O_3_ gas sensor can also respond to the target at this concentration level in the atmospheric environment. However, the concentration interpretation error of O_3_ is higher, because the concentration resolution of the O_3_ gas sensor is slightly poor. The annual average concentration of NO_2_ gas in 2020 was 11.16 ppb, indicating that the annual average concentration of NO_2_ gas was less than the resolution of the NO_2_ gas sensor (20 ppb), and its standard deviation is ±5.01 ppb, indicating that the concentration of NO_2_ gas is highly dispersive. Therefore, although the NO_2_ sensor may respond to the target gas at this concentration level, the concentration interpretation error of the NO_2_ sensor is more significant than that of the CO and O_3_ sensors due to the poor concentration resolution of the NO_2_ sensor.

### 2.2. Outlier Detection for Feature Selection

In the study, a local outlier factor (LOF) algorithm [39] filtered outlier data appearing in the data collection procedure. The problem of an insufficient cache occurred, given the long-term monitoring of the low-cost IoT devices in the environment. Therefore, the IoT devices were designed to reset regularly every 5 min. In the resetting process, conflicts between the Arduino Mega2560 embedded device, the NodeMCU WIFI chip, and the gas sensor occasionally appeared, resulting in mixed outliers in data uploading, which is defined as data loss. Data loss caused the features of gas concentration, temperature, and humidity to change rapidly in a short period of time and disturbed the data preparation. Therefore, we used a LOF algorithm to filter outliers generated by the data loss. The idea of the LOF algorithm is to quantify the density of each sample point and compare the density of the quantized sample point with the density of its neighboring points. Whether a sample point is an outlier depends on the degree of difference between the local density of the sample point itself and that of its neighboring reference points. If the local density is significantly different from its reference neighbor, the sample point is regarded as an outlier and vice versa. Applying this method to outliers can prevent drastic data changes due to data loss and effectively improve the quality of training data.

**Figure 3:**
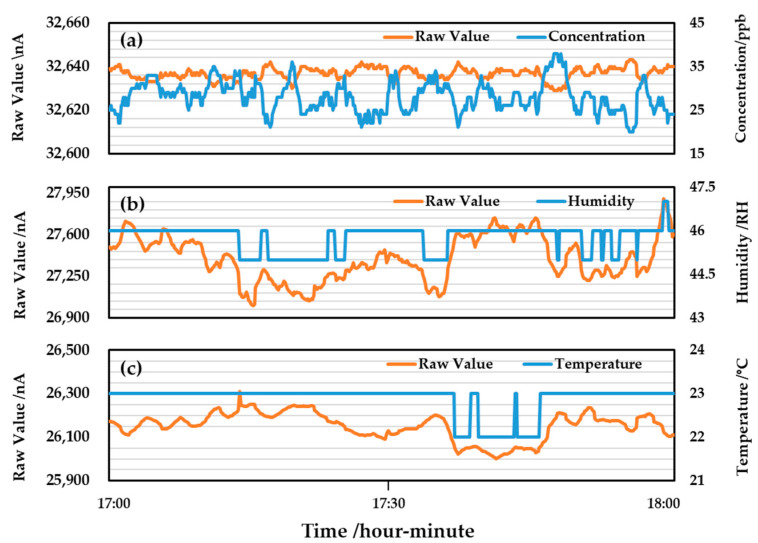
Raw values and corresponding physical quantities of (**a**) concentration, (**b**) temperature, and (**c**) humidity of the O_3_ gas sensor on February 1 2021 (17:00–18:00).

For example, the features collected by the O_3_ gas sensors are shown in Figure 3. Figure 3 contains the raw values (orange line) and corresponding physical quantities (blue line) of the concentration, temperature, and humidity. The raw values are the digital signals of the ADC converter (in nano amperes nA). According to the conversion formula given by the dealer, the raw values convert into the corresponding physical quantities. The physical quantity is the ppb value of O_3_ gas concentration, temperature (°C), and relative humidity (RH). The converted physical quantity is stored as an integer variable, which loses the data after the decimal point. Therefore, in feature selection, only the raw value of the gas sensor is retained, and the physical quantity obtained by the converted formula of the gas sensor is discarded.

### 2.3. Data Preprocessing—Normalization and Division

The datasets collected from the environment required processing before developing machine learning models. The variation ranges of the data collected by gas sensors are different, and thus normalization was adopted to avoid the unbalance effect of the essential features. Data were normalized using the MinMaxScaler equation, and the formula is expressed as:

(1) $$X_{norm.\;}=\frac{X-X_{max}}{X_{max}-X_{min}}\in \left[0,1\right]$$

where X_*max*_ is the maximum value of the data, X_*min*_ is the maximum value, and X is the original value. After the normalization, the X_*norm.*_ is scaled to the range [0, 1], and the trend properties remain.

The data collected from the environment is time-dependent with a sequence property. The commonly used random data shuffling is unsuitable for developing our machine learning models, because data leakage occurs if the sequential data is normalized after random shuffling. The shuffled normalized data for model training may contain the information of the testing dataset, such as the upper and lower limits, which can lead to over-optimistic training results for the offline model training.

**Figure 4:**
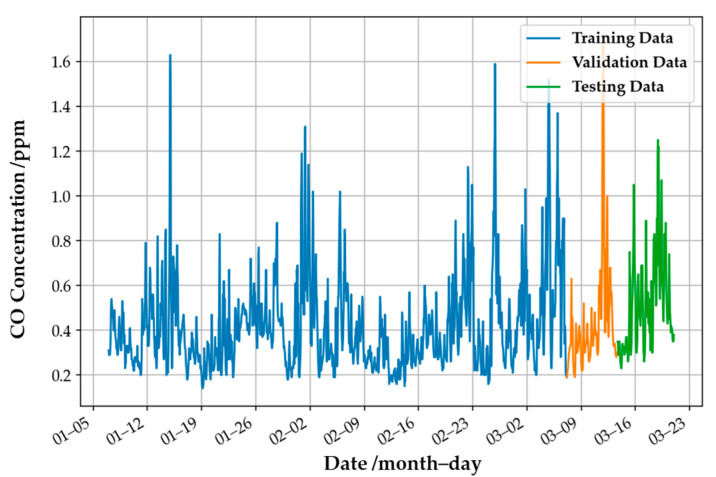
An example of the datasets for the training and testing phases. The CO gas concentration data were obtained from the EPA website.

This study divided the data into three parts: the training and validation datasets in the training phase and the testing set in the testing phases. As an example, Figure 4 shows these three parts for the CO gas concentration. The blue, orange, and green lines represent the training, validation, and testing data. These sets do not overlap each other in time series to avoid the problem of data leakages in the model development. The training dataset is for the model training, it is validation data to be used for the hyperparameters’ adjustment by minimizing the validation error, and the testing set is to test the actual performance of the target gas model. All datasets in this paper were treated using this method to develop a high-performance model.

## 3. Basics of Machine Learning

In machine learning, hyperparameters are parameters used to control the learning process. Proper hyperparameters can make the model converge to a better local minimum. Hyperparameters include the model hyperparameter, i.e., the model type, and the algorithm hyperparameters, including the layers, the number of neurons, and the learning rate. Since the data in this study belongs to time-series data, considering the property of the data, the Recurrent Neural Network (RNN) [21] is selected. RNN can be used to analyze time-series data and extract information between data through the gate unit inside the model so that the RNN has a breakthrough in processing time-series data.

### 3.1. Recurrent Neural Network

**Figure 5:**
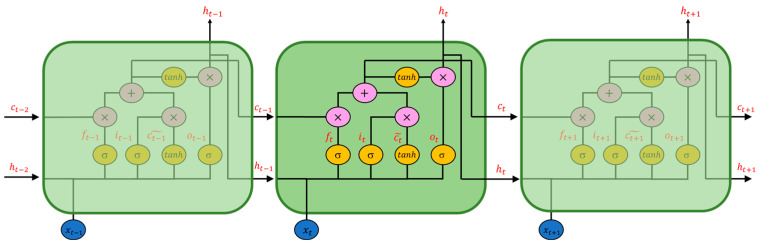
Structure diagram of LSTM.

The four types of RNNs used in the subsequent experiments are introduced in this section. The first RNN is the Long Short-Term Memory (LSTM) network. The LSTM comprises multiple memory cells, and each memory cell has three gate units: the forget gate, input gate, and output gate. LSTM controls the amount of hidden state information through the gated unit and improves the phenomenon that Simple-RNN is prone to, i.e., gradient explosion or gradient vanishment, when dealing with long time-series problems. Figure 5 shows a schematic diagram of the LSTM structure composed of three memory cell units, and the parameters are defined in the following equations.

(2) $$f_{t}=\sigma \left(W_{f}\cdot \left[h_{t-1},x_{t}\right]+b_{f}\right)$$

(3) $$i_{t}=\sigma \;\left(W_{i}\cdot \left[h_{t-1},x_{t}\right]+b_{i}\right)$$

(4) $$o_{t}=\sigma \;\left(W_{o}\cdot \left[h_{t-1},x_{t}\right]+b_{o}\right)$$

(5) $$\tilde{c_{t}}=tanh\;\left(W_{c}\cdot \left[h_{t-1},x_{t}\right]+b_{c}\right)$$

(6) $$c_{t}=f_{t}\cdot c_{t-1}+i_{t}\cdot \tilde{c_{t}}$$

(7) $$h_{t}=o_{t}\cdot tanh\left(c_{t}\right).$$

The forget gate is represented by $f_{t}$, the input gate is $i_{t}$, and the output gate is $o_{t}$. The index *t* presents the time step. Thus, $x_{t}$ and $h_{t}$ are the input and hidden states at the current moment. $\tilde{c_{t}}$ represents the updating value of cell state, and $c_{t}$ is the cell state at the current time. The forget gate, input gate, and output gate are activated by the sigmoid function and the updating value of the cell state by the hyperbolic tangent function. $W_{f}$, $W_{i}$, $W_{o}$, $W_{c}$ are the weight matrix of the forget gate, input gate, output gate, and cell state, respectively, while $b_{f}$, $b_{i}$, $b_{o}$, $b_{c}$ are the bias matrix. The forget gate $f_{t}$ controls the proportion of $c_{t-1}$, the cell state at the last time step, to be forgotten in the current cell state $c_{t}$; the input gate $i_{t}$ determines how much of the current updating value $\tilde{c_{t}}$ is needed; the output gate $o_{t}$ determines the proportion of the cell state $c_{t}\;$ to be used as an output and to obtain the hidden state $h_{t}\;$ of the memory cell at the current moment. For more information on LSTM, refer to Hochreiter and Schmidhuber [23].

**Figure 6:**
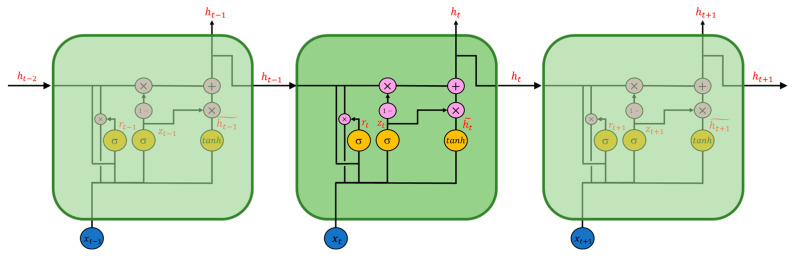
Structure diagram of GRU.

The second type of RNN is the Gated Recurrent Unit (GRU) network, a simplified version of LSTM. Each GRU memory cell has two gate units: the update gate and the reset gate. Compared with LSTM, GRU has fewer parameters, reducing the time spent in model training and the cost of hardware calculation. Figure 6 shows a schematic diagram of the GRU structure composed of three memory cell units, and Equations (8)–(11) define the operators.

(8) $$r_{t}=\sigma \left(W_{r}\cdot \left[h_{t-1},x_{t}\right]+b_{r}\right)$$

(9) $$z_{t}=\sigma \;\left(W_{z}\cdot \left[h_{t-1},x_{t}\right]+b_{z}\right)$$

(10) $$\tilde{h_{t}}=tanh\left(W_{h}\cdot \left[r_{t}\cdot h_{t-1},x_{t}\right]+b_{h}\right)$$

(11) $$h_{t}=\left(1-z_{t}\right)\cdot h_{t-1}+z_{t}\cdot \tilde{h_{t}}$$

In the above formula, $r_{t}\;$ represents the reset gate, $z_{t}\;$ the update gate, $\tilde{h_{t}}$ the updating value of the hidden state, and $x_{t}$ and $h_{t}$ are the input and hidden state at the current time, respectively. The reset gate and the input gate are activated by the sigmoid function and the updating value of the hidden state by the hyperbolic tangent function. $\;W_{r}$, $W_{z}$, $W_{h}$ are the weight matrix of the reset gate $r_{t}$, update gate $z_{t}$, and the updating value of the hidden state $\tilde{h_{t}}$, and $b_{r}$, $b_{z}$, $b_{h}$ are the bias matrix of $r_{t}$, $z_{t}$, and $\tilde{h_{t}}$, respectively. The update gate controls the ratio of the hidden state $h_{t-1}$ at the last time step (t−1) and the updating value of the hidden state $\tilde{h_{t}}$ at the current time; the reset gate $r_{t}\;$ resets the information of the hidden state $h_{t-1}$. More information on GRU is in reference [24].

**Figure 7:**
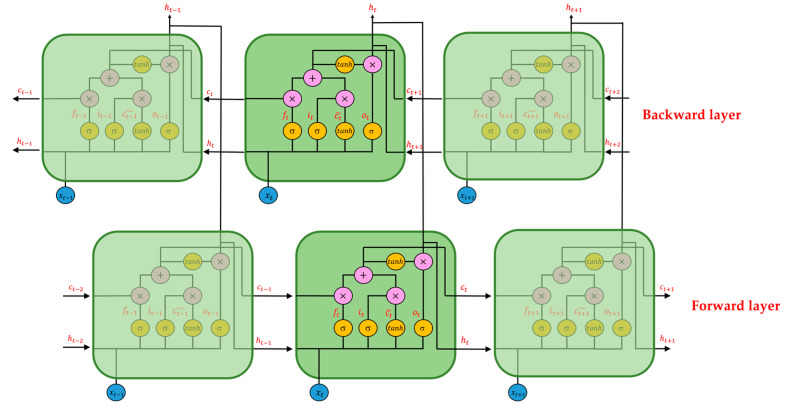
Structure diagram of Bi-LSTM.

**Figure 8:**
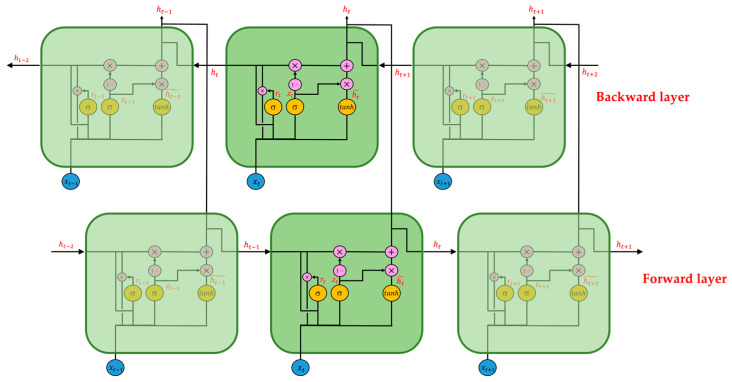
Structure diagram of Bi-GRU.

The third and fourth types of RNNs are the Bi-directional Long Short-Time Memory (Bi-LSTM) and Bi-directional Gated Recurrent Unit (Bi-GRU) networks, and their schematic diagrams are in Figure 7 and Figure 8.

Compared with LSTM and GRU, Bi-LSTM and Bi-GRU improve time flow. In the forward-transmission RNN, a new backward RNN is built, and the time flow of the two is precisely opposite. Therefore, for Bi-RNNS, there are two RNNs with opposite time flows, namely the forward layer and the backward layer; the information of memory cells corresponding to the same time is provided by the output value of both the forward and backward RNNs. Therefore, compared with the general form of RNN, Bi-RNN can consider the information of the whole time series and make full use of the context of the time series. For more information on Bi-RNN, refer to Schuster and Paliwal [25].

### 3.2. Construction of Model

**Figure 9:**
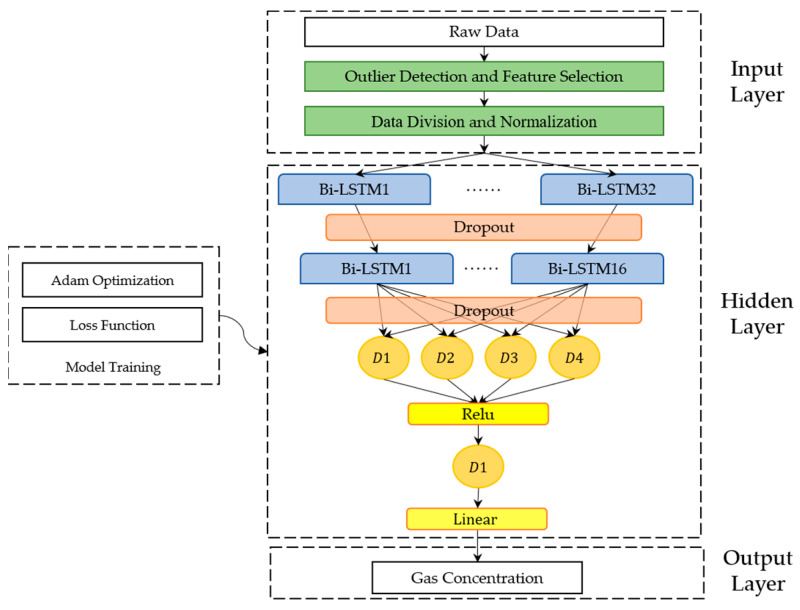
Structure diagram of the gas model.

In this study, the machine learning models used to detect gas concentration include three parts: the input layer, the hidden layer, and the output layer, as shown in Figure 9. The input layer preprocesses raw data using the LOF algorithm and the MinMaxScaler method. The hidden layer comprises the recurrent neural layer (the blue box in Figure 9) and the fully connected layer (the orange circles, referred to as the FC layer). The Bi-LSTM memory cells (hereafter referred to as the BiL layer) are shown in the recurrent neural layer as an example. The number of layers and neurons varies according to the type of gas model; the dense layer number is set as two. The weight of the hidden layer of the model is optimized by the Adam optimizer [40] with a mean square error (MSE) loss function so that the loss function converges to a better local minimum. The output layer receives the information from the previously hidden layer and calculates the detected gas concentration through a linear activation function. In our study, the three-layer models are flexible enough to develop high-performance machine learning models.

The concentration-detecting models use a loss function and an evaluation function. The MSE loss function in the hidden layer is defined as:

(12) $$\mathrm{MSE}=\frac{1}{n}\sum_{i=1}^{n}{\left(y_{predict\left(i\right)}-y_{true\left(i\right)}\right)}^{2},\;$$

where $y_{predict\left(i\right)}$ is the model gas concentration output value, $y_{true\left(i\right)}$ is the actual value, and n is the total sample number. The variance of each sample is obtained by subtracting the model output value and the corresponding actual value by square. Then, the MSE of the model can be obtained by summing up the variance values of all samples and averaging them. The evaluation function in this study is Mean Absolute Percentage Error (MAPE), defined as:

(13) $$\mathrm{MAPE}=\frac{100\%}{n}\overset{n}{\underset{i=1}{\sum }}\left|\frac{y_{predict\left(i\right)}-y_{true\left(i\right)}}{y_{true\left(i\right)}}\right|.\;\;$$

After subtracting the model output value and the corresponding actual value of each sample, then dividing the corresponding true value and taking the absolute value, the MAPE of each sample is obtained. MAPE defines a dimensionless error, and thus it is suitable to use to measure the differences between the model output and the actual values, even under different numerical magnitudes.

## 4. Development of Ensemble Models

Ensemble models consisting of different RNNs were developed to detect target gas concentrations in this study. Traditionally, developers use the validation dataset and take the evaluated index to obtain the best machine learning model. The steps include: 1. a series of hyperparameter tests by the training data; 2. evaluating the performance by the validation data; and 3. selecting the model hyperparameter which shows the best evaluation in the last step. The optimal model selected by the above procedures can achieve the best performance in the validation set, but some disadvantages exist. First, the procedure is time- and labor-consuming, but only one, the optimal hyperparameter configuration, is selected. The other models that result from the hyperparameter optimization are abandoned. Next, the features of the validation dataset can not guarantee consistency with those of the future new data. The model with the best performance of the validation-run set may not have the best performance while applied to the new data in the future. Thus, improvement by using an ensemble model was proposed to enable the modification of the model and learn the generalizability of future data.

Developing ensemble models includes optimizing hyperparameters, comparing memory cells, and ensembling and retraining the best model. The machine learning programs were based on Python3.8, Tensorflow-gpu 2.4.0, and execution on graphics cards of NVIDIA Titan XP and NVIDIA RTX 3080. Details are in the following subsections.

### 4.1. Optimization of Hyperparameters

In machine learning, hyperparameters include the model hyperparameters and the algorithm hyperparameters. Model hyperparameters, such as the number of layers, neurons, and input features, affect the model’s best performance. Algorithm hyperparameters include the selection of the optimizer, learning rate, batch size, etc., which significantly affect the convergence and training time of the model. A series of hyperparameter optimizations was processed in the study. We first optimized the model hyperparameter to determine the basic architecture of each gas model. Then, the algorithm hyperparameters were optimized so that each gas model could shorten the training time and converge to a better local minimum.

The model hyperparameters of a single weak model for detecting a specific gas were determined firstly. Configurations of the model for a single-gas detection (CO, O_3_, and NO_2_) are shown in Table 4. We compare the influence of the number of BiL layers on the performance of each gas model, which is one to three layers, respectively. Each layer has a specific number of neurons, which is a power of 2, as shown in the brackets. Finally, the number of BiL layers of the CO model was set to two, and the number of BiL layers of the O_3_ model and the NO_2_ model was set to three.

Next, we compared the performance of the model validation set with the number of input features to determine the number of input features for each gas model. The input feature contains the raw values from the gas sensors (gas concentration, humidity, and temperature). The MAPE of the CO gas model is 16.31% when it is trained with the dual gas features of CO and O_3_ and 17.35% when trained with the single-gas feature of CO. The performance of the dual-gas features is improved by 1.04%. The MAPE of the O_3_ gas model was 36.98% after training with O_3_ and NO_2_ dual gas features and 41.67% after training with O_3_ single gas features. The performance using the dual-gas feature is 4.7% higher than that of the single-gas feature; the reason is that the O_3_ gas sensor is disturbed by NO_2_ gas (as mentioned in Section 2.1). Therefore, adding the O_3_ gas feature can significantly improve the performance of the validation set of the O_3_ gas model. The MAPE of the NO_2_ gas model was 86.27% after training with O_3_ and NO_2_ dual gas features and 68.05% after training with NO_2_ single gas features. The performance of the single-gas feature is 18.22% higher than that of the dual-gas feature; the reason is that the NO_2_ gas sensor is less disturbed by O_3_ gas similarly. Therefore, adding the O_3_ gas feature will reduce the validation set performance of the NO_2_ gas model. Through the above experiments, we determined the model hyperparameters of the gas model, including the basement architecture and input features. Next, the algorithm hyperparameter for each gas model will be optimized.

The algorithm hyperparameters, e.g., the batch size and the dropout layer coefficient, are optimized to reduce the model’s training time and improve the model’s convergence. The batch size is the number of samples used for training once. A larger batch size can shorten the training time of the model, but the variance between batches is slight when calculating the gradient of each batch in reverse. Therefore, the gradient obtained from each batch varies little, and the lack of gradient randomness tends to fall into a poor local minimum. Smaller batches require a longer calculation time for each iteration, which prolongs the training time of the model and increases the time cost of model tuning. However, compared with the large batch, the small batch has the advantage of gradient randomness, resulting in it converging better to the local minimum. In summary, choosing an appropriate batch size is necessary to balance the training time and convergence of the model.

**Figure 10:**
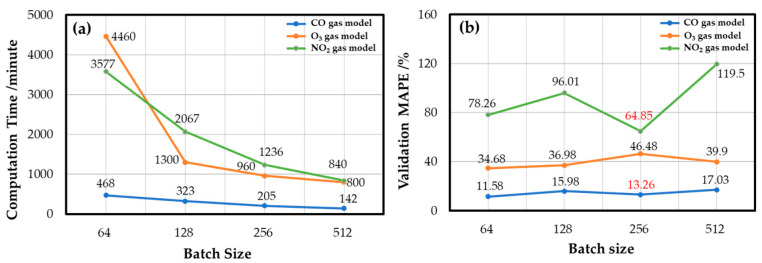
Batch size effect of each gas model: (**a**) computation time and (**b**) the convergence as the validation MAPE.

Figure 10 shows the experimental results of the effects of the batch size in developing the gas models. Although the batch size of 64 achieved the best convergence, it took twice the computation time as long as the batch size of 256, and the performance difference between the two was only 1.68%. Finally, the batch size of 256 was selected as the best batch size configuration for the CO gas model. The decision of O_3_ gas and NO_2_ gas models also considered the time cost, and the final batch sizes were 128 and 256, respectively.

**Figure 11:**
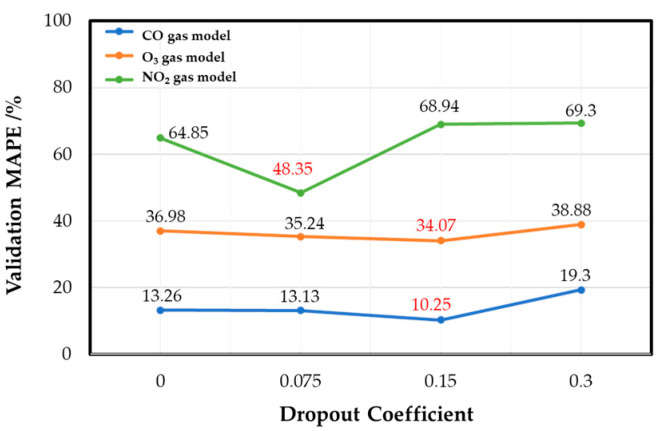
Dropout coefficient effect of each gas model.

After this, the dropout layer coefficients of each gas model were determined. The dropout layer is a method used to improve model overfitting by shielding a certain percentage of neurons in each epoch, so that model training does not rely too much on specific neurons for training and prevents model overfitting [41]. By adjusting the coefficient of the dropout layer appropriately, the overfitting phenomenon of each target gas model on the training set can be effectively alleviated, and the performance of the verification set of each target gas model can be effectively improved. Figure 11 shows that, when the dropout coefficient is 0.15, the CO gas and the O_3_ gas models have the best validation set performance, with 10.25% and 34.07% MAPE, respectively. The NO_2_ gas model has the best validation performance when the dropout coefficient is 0.075, with 48.35% MAPE.

By optimizing the model hyperparameters and algorithm hyperparameters, the single weak model of each gas was trained. Subsequently, multiple single weak models were created for the ensembles by replacing the memory cell units of different types to improve the poor generalization of the single weak model on the new data.

### 4.2. Comparison of Memory Cells

**Figure 12:**
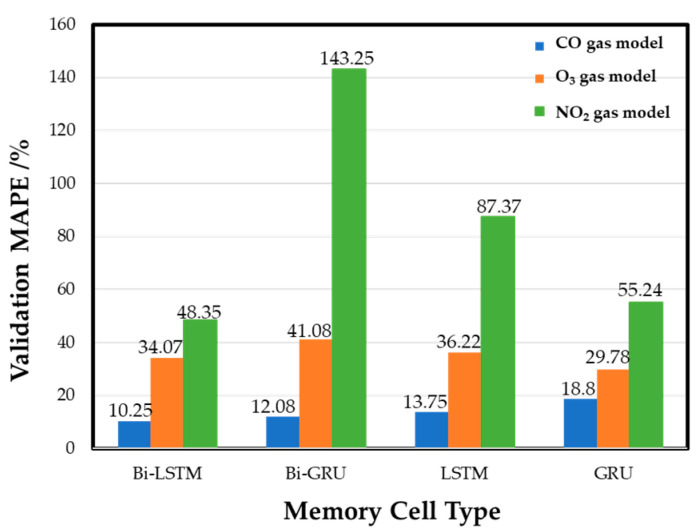
Effects of different memory cells on gas models.

The memory cell used in the model is a vital model hyperparameter to be discussed. The recurrent neural layer uses the Bi-LSTM memory cell unit in the last section. More types of different memory cell units, including Bi-GRU, LSTM, and GRU, are compared based on their performance in the validation set testing. Figure 12 shows the experimental training results of different memory cells in each gas model. The results show that Bi-LSTM is the best cell for the CO gas model, with a MAPE of 10.25%. The other MAPE values are 12.08% for Bi-GRU memory cells, 13.75% (LSTM), and 18.8% (GRU). The performance of the O_3_ gas model is best (29.78% MAPE) when GRU is used, and the MAPE values are 34.07%, 41.08%, and 36.22% for the Bi-LSTM, Bi-GRU, and LSTM cells, respectively. In the NO_2_ gas model, Bi-LSTM shows the best performance in the validation set, and the MAPE is 48.35%. The MAPE values obtained by other memory cell training models are 143.25% (Bi-GRU), 87.37% (LSTM), and 55.24% (GRU), respectively. The results show that the gas models trained by different memory cells have various performances in the validation set. Basically, the commercial sensor’s native resolution limits the performance. The NO_2_ sensor has the lowest ratio of the average annual concentration to resolution, and thus the MAPE of the NO_2_ sensor model is always higher than the models of the other two gases. Compared with the CO gas model and the O_3_ gas model, the NO_2_ gas models have the most considerable performance variation while different memory cells are used. Subsequently, by integrating each gas model trained by four different memory cells, an ensemble model can be retrained to be the best model for gas concentration detecting.

### 4.3. Ensemble Models to Obtain the Best Model

**Figure 13:**
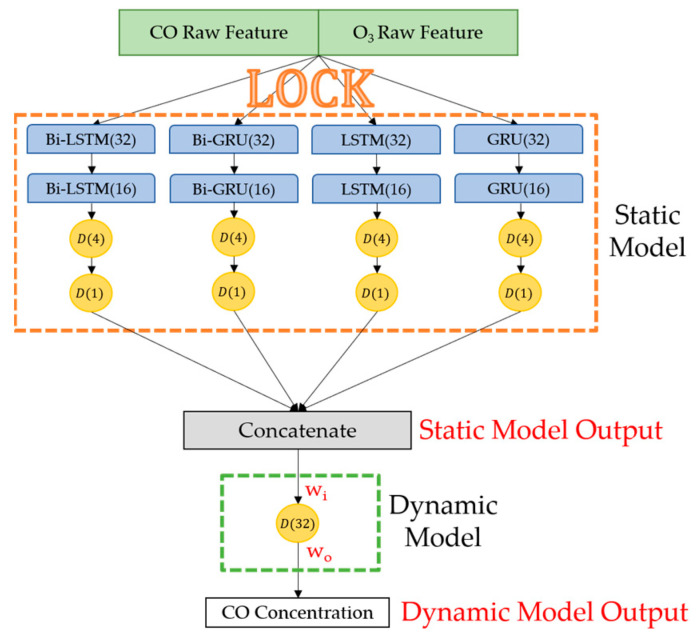
An example of schematic diagrams of gas ensemble model: CO gas ensemble model. Weight coefficients w_i_ and w_o_ change periodically through the further model retraining procedure.

Ensemble models are proposed in this study to reuse all the single weak models trained in the last steps. Figure 13 shows schematic diagrams of the ensemble models for CO gas; models for detecting O_3_ and NO_2_ gas are constructed in the same way. The orange dashed line in Figure 13 indicates the four types of recurrent neural models trained in Section 4.2. These recurrent neural models are integrated, and their parameters inherited from the last step are frozen in the ensemble model; thus, it is named a static model. The green dashed line in Figure 13 highlights a fully connected neural network responsible for receiving output values and training data from the static model and then determining their parameters through backpropagation. Since the weight coefficients (w_i_ and w_o_ in Figure 13) will change in the further retraining procedure, this NN is named the dynamic model. The dynamic model learns the deviation relation of different RNN models through retraining, summarizes the target gas concentration calculated by the static model, and outputs the final summarized target gas concentration value.

**Figure 14:**
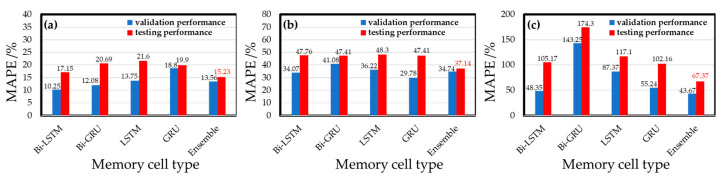
Performance differences between single gas model and ensemble gas model of (**a**) CO gas model, (**b**) O_3_ gas model, and (**c**) NO_2_ gas model.

The performance values of the ensemble model and every single weak model for three target gases are shown in Figure 14. Considering the CO gas models, in the training phase, the validation set testing of the ensemble model is not optimal, with a MAPE of 13.56%. The single models using Bi-LSTM and Bi-GRU memory cells have better MAPE values of 10.25% and 12.08%. However, in the testing phase, where the new data (i.e., testing dataset) were used, the 15.23% MAPE of the ensemble model is the best one in all models. The results show that all the CO single weak models have significantly lower performance values in the testing set than in the validation set. The Bi-LSTM single model, which has the best performance in the validation set, is not globally optimal for the test set, indicating that the single weak model has poor applicability to the new data. The ensemble model maintains a certain model performance on the new data and effectively improves the model’s generalizability to the new data.

A similar result was obtained from the experiments using O_3_ gas models. The ensemble model’s validation performance (34.74% MAPE) for O_3_ gas is not the best one compared to the other four single weak models, while the performances of the single-memory-cell model using GRU and Bi-LSTM were MAPEs of 29.78% and 34.07%, respectively. However, in the testing phase, all models examined new data (i.e., test dataset) for testing, and the integrated model had the best MAPE (37.14%) again. The O_3_ gas ensemble model is more applicable and keeps the model’s generalizability to new data.

In the tests of the NO_2_ gas models, the ensemble model has the best performance in both the validation and testing sets, and the MAPE values were 43.67% and 67.37%, respectively. The performance of the NO_2_ ensemble model decreases less than that of the single weak model in the test set compared with that of the single weak model in the validation set, indicating that the NO_2_ ensemble model is better than that of the single weak model in the application of new data. However, there is still room for improvement compared with the CO gas and O_3_ gas ensemble models.

It can be summarized that the static model part of the ensemble models contains the fundamental properties of the gas sensors found by the optimized single weak models. Further, the dynamic model part is a combination of the calibrated models and thus can achieve better performance by tuning weight coefficients. According to these experimental results of different gas ensemble models, the model deviation of a single-memory-cell model was effectively offset by integrating more types of recurrent neural models. Thus, ensemble models can have a better generalizability for handling individual differences in the commercial sensors of the same module; therefore, the ensemble model was chosen as the best model for gas concentration detection.

### 4.4. Ensemble Model Retraining

In the previous section, the ensemble model had the best performance for each gas, but further tests observed a decayed performance while more new data were input. The reason is that the data used to train the ensemble RNN models were collected from January 5 to March 23, containing only a partial property in a whole year. Thus when the new data collected from March 23 to April 14 was input into the model, the performance became unstable, because the atmospheric conditions changed across different seasons. Therefore, we propose a periodical retraining procedure. The periodic retraining procedure regularly updates the dynamic model’s weights to conform to the deviation of the characteristic distribution of the new dataset collected in the atmospheric environment in each period. It extends the life cycle of the integrated gas models.

**Figure 15:**
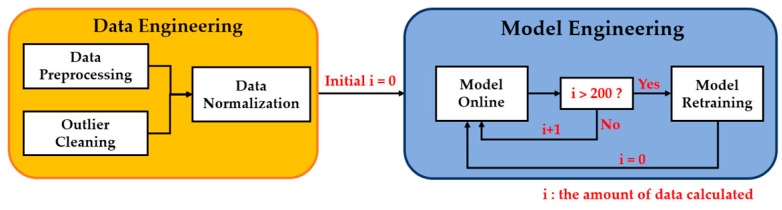
Retraining flow chart for the ensemble gas model.

The flow chart of an optimal gas model is set out in Figure 15. Data engineering is the first step in dealing with a new dataset, including preprocessing, outlier cleaning, and normalization, as mentioned in Section 2. The second step, model engineering, contains a recursive work—model online, performance monitoring, and model retraining. We obtained the best ensemble model through a training series, as shown in Section 4.1, Section 4.2 and Section 4.3. Then, the model was activated to calculate gas concentrations; this model was named *model online*. Under regular monitoring, the model’s performance declined with time; therefore, the program counts on the amount of data that the model has calculated and decides whether to retrain the dynamic model. While the amount of data calculated by the model reached two hundred, model retraining was triggered in the study. The testing dataset became a new training dataset in the retraining procedure. The upper and lower limits of the scaling scale of the new training dataset were consistent with the original training dataset to ensure the consistency of the new and old datasets. We used the new training dataset to train the dynamic model again and then updated the weight coefficients (as shown in Section 4.3). By periodically updating the dynamic model’s weight coefficients, the gas model’s performance at each stage is stabilized and extends the gas model’s life cycle.

**Figure 16:**
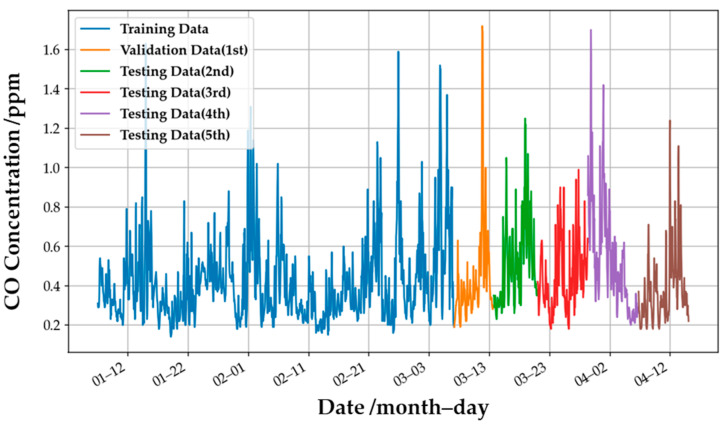
CO gas concentration at different periods measured by the EPA’s monitoring station.

The ensemble model for CO gas was used to demonstrate the performance of the retrained model. Figure 16 shows the actual CO concentration values provided by the EPA, and the lines in different colors present the definition of the dataset. The initial single weak models were trained by the training dataset (the blue line, which contains 1500 pieces of data), and the ensemble model for CO was defined. Then the weights of the dynamic model are defined by updating the weights of the original model according to the newly added data in each period (i.e., the orange, green, red, purple, and brown lines). The amount of data for each period is 200, and the initial learning rate of the model is 0.1 times that of the original static model. The retraining method of the O_3_ and NO_2_ gas models is the same as that of the CO gas model: use the previous period’s data as new training data, and retrain the model through the new data of the previous period to improve the generalization ability of the retrained model in the next period.

**Figure 17:**
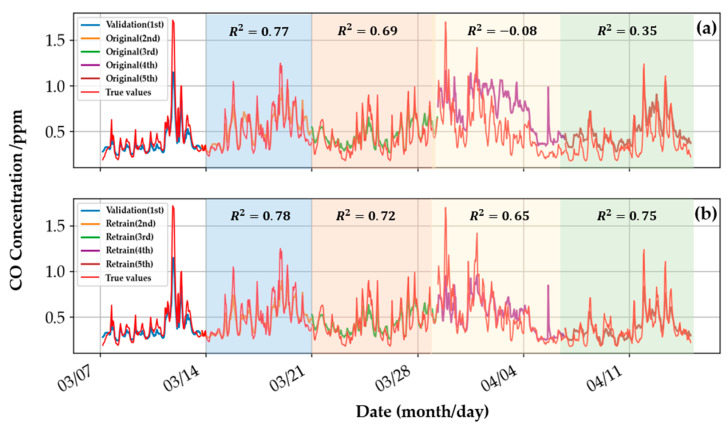
The performance comparison of the (**a**) original model and the (**b**) retrained model of the CO gas ensemble model at different periods. The true values of concentrations were from the EPA’s database.

Figure 17 shows the actual CO concentration, the original model’s output values, and the retrained model’s outputs. The weight coefficients in the original ensemble model are unchanged after being defined in the training phase. Figure 17a shows the significant differences between the output concentrations and the actual values. The performance was estimated using the residual sum of squares of the linear regression model, i.e., the R^2^ value. In the fourth interval, the R^2^ value of the original CO gas model is negative, hinting at the failure of the linear regression model, and the model’s overall performance is not stable enough to handle changes in new data. The results of the O_3_ and NO_2_ gas models are similar, and the results are summarized in Table 5. The retrained model updated the gas model through the last period’s data and dynamically corrected the concentration interpretation of the static model in each period. Thus Figure 17b shows a smaller difference in each period. The retrained model has an average R^2^ of 0.73 over the four intervals. As shown in Table 5, the long-term average R^2^ of the four intervals of the retrained O_3_ and NO_2_ gas ensemble model are 0.51 and 0.37, respectively, which are better than the results of the original ensemble models.

Compared with the original model without retraining, the sensor performance of the retrained model is much more stable in the different periods. The retraining procedure can update the parameters of the dynamic model in the ensemble model, meaning that the machine learning model with regular retraining is a potential solution to calibrate sensors deployed in different environments (area or season). Thus, the model’s life cycle is effectively prolonged.

Furthermore, the contribution of each static model to the final output value of the ensemble model was estimated. The degree of the gradient contribution was used, which was obtained by differentiating the output of the dynamic model with respect to that of the static model as follows:

(14) $$\mathrm{gradient}\;\mathrm{contribution}=\frac{\partial {\mathrm{Dynamic}\;\mathrm{model}}_{\mathrm{output}}}{\partial {\mathrm{Static}\;\mathrm{model}}_{\mathrm{output}}}$$

**Figure 18:**
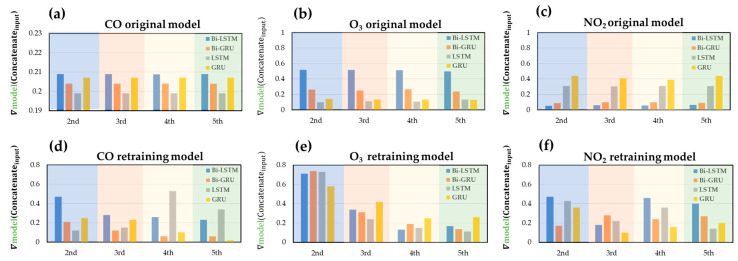
The gradient contributions of the static models in the original ensemble model for (**a**) CO, (**b**) O_3_, and (**c**) NO_2_ gases and in the retrained ensemble models for (**d**) CO, (**e**) O_3,_ and (**f**) NO_2_ gases.

The gradient contribution of every data point in each interval was calculated, summed, and averaged, and the results are shown in Figure 18. In the original gas models, the gradient contribution of each static model to the dynamic model is unchanged in all periods. The original gas model interprets gas concentration on the new data with the same gradient contribution in each period; the model cannot be dynamically adjusted with time, resulting in poor model performance and generalizability on the new dataset. Compared with the original gas model, the retrained gas model uses the new data of the previous period to update the weight coefficients of the dynamic model. Thus, the retrained gas model adjusts the gradient contribution of the static model and corrects the static model output in each period; the improved concentration estimations were observed in Figure 17 and Table 5. The retraining procedure provides the ensemble models better generalizability on new data.

### 4.5. Feasibility of Onsite Gas Sensing

**Figure 19:**
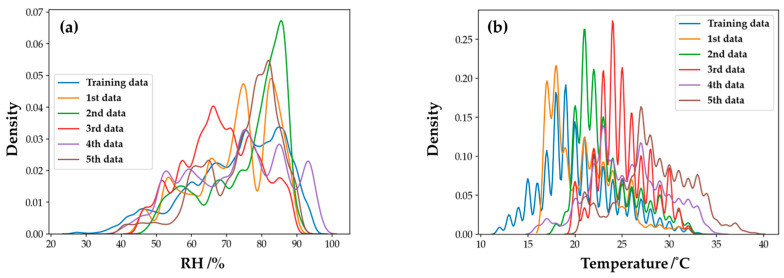
Distribution of (**a**) humidity and (**b**) temperature in each period.

This study discussed the feasibility of using IoT gas sensors in a natural atmospheric environment. According to the Kernel Density Estimation (KDE) [42] of humidity and temperature, as shown in Figure 19, our sensors were in a rapid-change humidity and temperature environment instead of a constant temperature/humidity chamber in a laboratory. Under these varying conditions, our ensemble model can still maintain a relatively stable performance. The ensemble machine learning model can apply IoT sensors to achieve accurate air pollution detection.

A comparison of the sensing performance and equipment cost of the gas sensing equipment of other manufacturers is shown in Table 6 (reference source: AQ-SPEC [43]). The R^2^ performance of our low-cost IoT device with AI assistance in all target gases is slightly lower than that of other gas-sensing devices, but the gap is not significant. Considering the cost of a large number of deployed gas detection equipment, the low-cost IoT device in this study is far less expensive than other brands of gas detection equipment. Based on cost performance advantages, more low-cost IoT devices such as the one developed in this study can be deployed to improve the spatial density information of CO, O_3_, and NO_2_ gases.

## 5. Conclusions

This paper studied ensemble models of RNN for onsite gas concentration detection using low-cost commercial sensors. IoT sensing devices for CO, O_3_, and NO_2_ were designed, fabricated, and then deployed in the field to monitor atmospheric air conditions. The time-sequence data of concentration, temperature, and humidity were collected for three months. Single weak RNN models for the three target gases were developed first, and then the ensemble models combining four types of RNN models were defined and studied. Results showed that the ensemble models improved the sensing performance for all gases. The results show that integrating four types of RNN models can significantly improve the performance in the testing set, showing a better result than any single RNN model. The static model part of the ensemble models contains the fundamental properties of the gas sensors, and the dynamic model part is a combination to achieve better performance. Thus, the ensemble model has a better generalizability for the commercial sensors for gas concentration detection.

Furthermore, a retraining procedure was designed as the optimal model to maintain stable model performance and prolong the life cycle. The performance of the original model without retraining is volatile in different periods, while the retraining model can solve this problem well. The periodic retraining procedure can update the parameters of the dynamic model in the ensemble model, meaning that the trained machine learning models can be easily applied while the sensors are deployed in a different environment (area, season). The results showed that the long-term average determination coefficient (R^2^) of the CO gas model reaches 0.73, it reached 0.51 for the O_3_ gas model and 0.37 for the NO_2_ gas model. The performance is still limited by the native sensitivity and the target selectivity. However, with the help of our ensemble models, these sensors have a specific correlation with the actual concentration announced by the EPA. The results promise accurate air pollution detection feasibility using commercial gas sensors in natural changing temperatures and humidity environments.

## Figures and Tables

**Table 1:** Performance characteristics of each sensor [37].

Target Gas	CO	O_3_	NO_2_
Module	DGS-CO 968-034	DGS-O_3_ 968-042	DGS-NO_2_ 968-043
Range	0 to 1000 ppm	0 to 5 ppm	0 to 5 ppm
Resolution	0.1 ppm	20 ppb	20 ppb

**Table 2:** Cross sensitivity of each sensor [37].

Applied Gas	Typical Response		
	CO Sensor	O_3_ Sensor	NO_2_ Sensor
CO	1:1	-	-
O_3_	1:<0.2	1:1	1:<0.1
NO_2_	-	1:1	1:1

**Table 3:** Average annual concentrations of CO, O_3_ and NO_2_ in Taiwan in 2020 [38].

Type of Gas	Average Annual Gas Concentrations	Standard Deviation
CO	0.35 ppm	±0.15 ppm
O_3_	30.9 ppb	±3.95 ppb
NO_2_	11.16 ppb	±5.01 ppb

**Table 4:** Performance differences of target gas models with different model structures.

Target Gas	Model Structure	Input Feature	MAPE (Validation)
CO	BiL(32)–BiL(16)–NN	CO + O_3_	16.31%
		Only CO	17.35%
O_3_	BiL(64)–BiL(32)–BiL(32)–NN	O_3_ + NO_2_	36.98%
		Only O_3_	41.67%
NO_2_	BiL(64)–BiL(32)–BiL(32)–NN	O_3_ + NO_2_	86.27%
		Only NO_2_	68.05%

**Table 5:** Comparison of performance between original model and retrained model.

Testing Dataset	R^2^					
	Original			Retrained		
	CO	O_3_	NO_2_	CO	O_3_	NO_2_
2nd	0.77	−0.22	−0.22	0.78	0.54	0.36
3rd	0.69	−0.40	−4.00	0.72	0.47	0.37
4th	−0.08	0.59	0.13	0.65	0.58	0.34
5th	0.35	0.27	−0.04	0.75	0.46	0.40
Average R^2^	0.43	0.06	−1.03	0.73	0.51	0.37

**Table 6:** Comparison of the sensing performance and equipment cost of the gas sensing equipment of other manufacturers.

Sensor Image	Model Name	Cost (USD)	Field R^2^		
			CO	O_3_	NO_2_
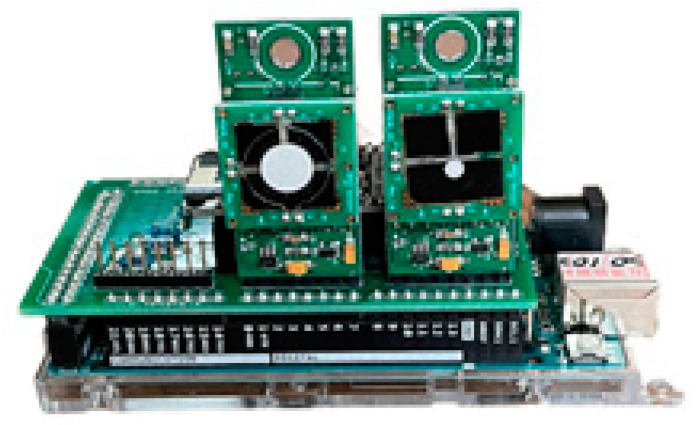	Low-cost IoT Device	$200	0.65 to 0.78	0.46 to 0.54	0.34 to 0.4
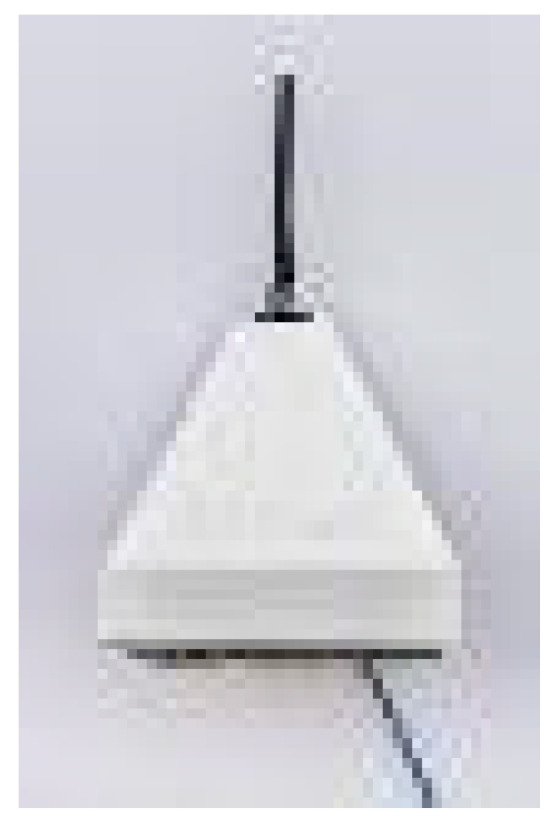	AQMesh V5.1	$7800	0.9 to 0.94	0.62 to 0.74	0.49 to 0.54
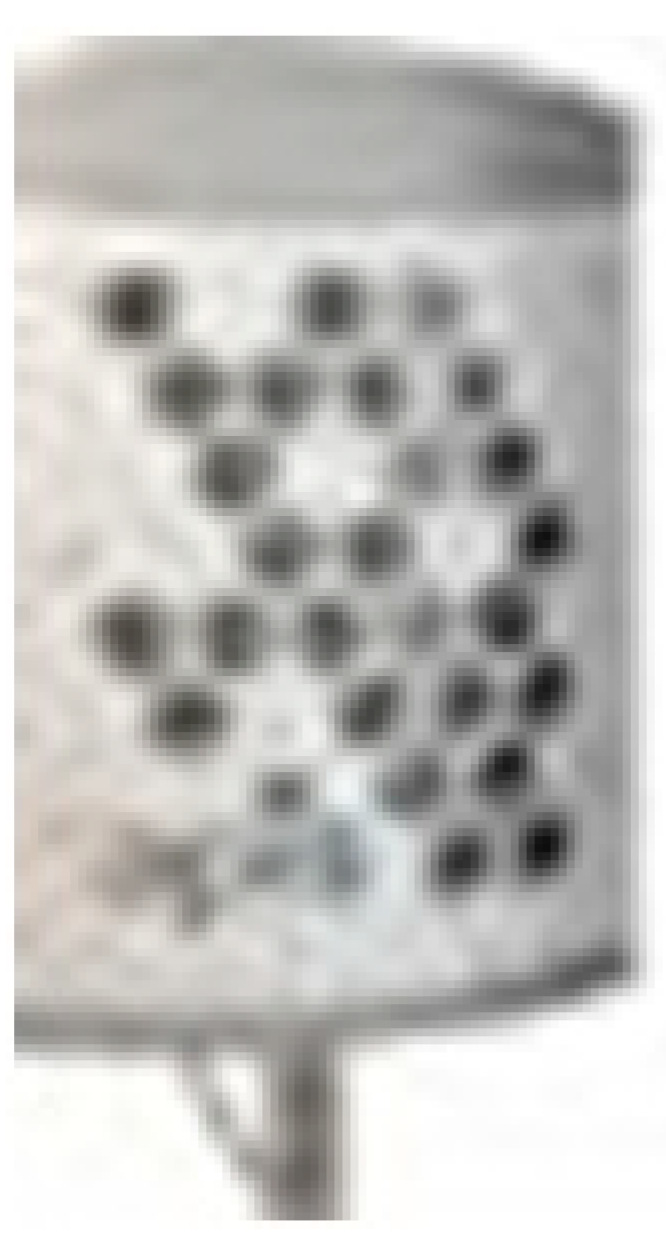	APIS	$4995	0.87 to 0.9	0.73 to 0.83	0.3 to 0.44
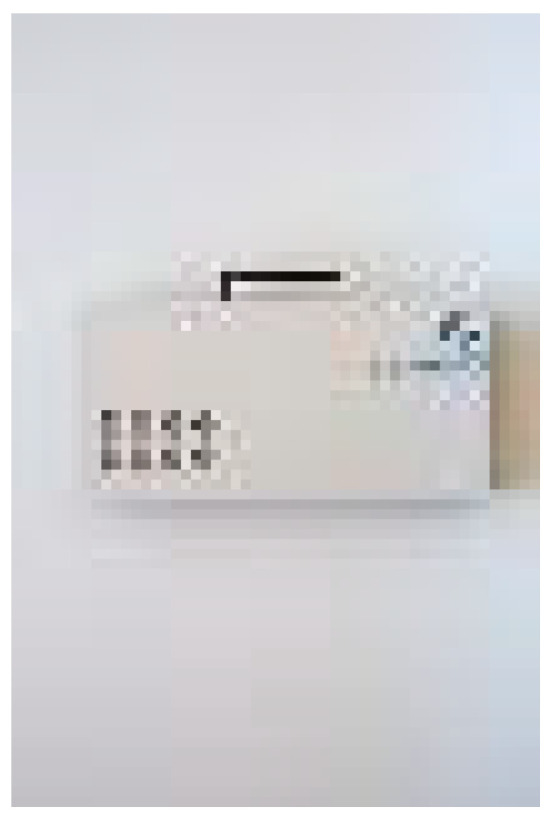	Igienair (Zaack AQI)	$3000	0.84 to 0.87	0	0.53 to 0.58
